# Comparative Assessment of Enamel Microhardness Using Various Remineralising Agents on Artificially Demineralized Human Enamel

**DOI:** 10.7759/cureus.30281

**Published:** 2022-10-13

**Authors:** Rikki Deswal, Navneet Kukreja, Swati Chhabra, Shivangi Trivedi, Ankita Sharma, Anamika Thakur

**Affiliations:** 1 Department of Conservative Dentistry and Endodontics, Eklavya Dental College and Hospital, Kotputli, IND; 2 Department of Conservative Dentistry and Endodontics, Maharishi Markandeshwar (MM) College of Dental Sciences and Research, Ambala, IND

**Keywords:** remineralisation, microhardness, enamel, deminerlisation, dental caries

## Abstract

Introduction:Various remineralizing agents can be used to remineralize initial carious lesions.

Aim: The study aims to compare and evaluate the remineralizing efficiency of Remin Pro (VOCO GmbH, Cuxhaven, Germany), tricalcium phosphate, and HealOzone (CurOzone USA Inc., Ontario, Canada) by measuring the microhardness of enamel.

Materials and method: Forty-five mandibular premolars were collected and divided into three groups (A, B, and C). After sectioning mesiodistally, they were subdivided into the control and test groups. The test group was further subdivided into demineralized (A_2a,_ B_2a, _and C_2a_) and remineralized (A_2b,_ B_2b, _and C_2b_) groups. All test group samples were demineralized by immersing in demineralizing solutions for 24 hours. Afterwards, A_2b,_ B_2b,_ and C_2b_ samples were remineralized by remineralizing agents (Remin Pro, tricalcium phosphate, and HealOzone) for three minutes (twice a day) for 14 days, and then Vickers microhardness testing (VHN) was performed.

Result: The microhardness values of the demineralized group were lower compared to the samples of the control groups. In the remineralized group, the mean microhardness values were maximum for HealOzone (293.22 kgmm^-2^), followed by Remin Pro (287.5660 kgmm^-2^) and then tricalcium phosphate (282.4660 kgmm^-2^).

Conclusion: The application of remineralizing paste proved potent in improving the remineralization in the demineralized enamel surface.

## Introduction

Dental caries is a widespread disease that leads to demineralization, cavitation, pain, and discomfort, causing compromised aesthetics and functional limitations [1]. Caries is not simply a unidirectional, continuous proceeding of the mineral phase to demineralization; instead, it is a cyclic process of intervals of demineralization and remineralization in between. However, cavitations occur when the demineralization process predominates [2].

The demineralizing action at the advancing front of the lesion causes mineral loss at a certain depth below the surface of the enamel, accompanied by acidic ion transport to the advancing front of the lesion from the plaque and mineral ions towards the plaque. The remineralization process occurs under near-neutral physiological pH whereby calcium and phosphate mineral ions are re-deposited within the demineralized tooth structure from plaque and saliva and from newer hydroxyapatite crystals, which are larger and much more resistant to acid dissolution [3].

The advancements in the domain of caries research have coaxed knowledge of the disease course [1]. The early diagnosis of these incipient lesions and the focus on prevention play an important role in managing incipient carious lesions [4].

Various materials can initiate the remineralization process such as casein phosphopeptides, sugar substitutes, calcium, sodium phosphosilicate, ozone, hydroxyapatite, self-assembling peptide, and resin infiltrates to aid in the mechanism of remineralizing the tooth structure [5]. Tricalcium phosphate, known as hybrid material, is a combination of beta-tricalcium phosphate with sodium lauryl sulfate/fumaric acid, which effectively increases the efficiency of fluoride in remineralization by making calcium, phosphate, and fluoride accessible to the tooth surface, thereby enhancing mineral growth and strengthening the tooth structure [6,7]. Moreover, Remin Pro (VOCO GmbH, Cuxhaven, Germany) is a remineralizing water-based cream, comprising hydroxyapatite, fluoride, and xylitol. It acts by filling up eroded enamel, obliterating dentinal tubules with xylitol. It possesses antibacterial properties and strengthens the weakened tooth structure [8]. It has been suggested for the management of dentinal hypersensitivity, as well as incipient lesions [9].

A newly introduced remineralizing agent, HealOzone (CurOzone USA Inc., Ontario, Canada) is used in preventing the progression of caries by effectively killing the cariogenic bacteria in the lesions and facilitating the remineralization process [10,11].

Various techniques are employed for assessing the demineralization or remineralization of enamel or dentin such as microradiography, iodine absorptiometry, polarized light, iodide permeability, light-scattering, and wet chemical analysis. In this study, Vickers hardness number (VHN) testing was used to detect the microhardness in the tooth surface because the pyramid-shaped indent obtained is accurate in measurement and can detect visually and digitally [12].

Hence, the aims and objects of this in-vitro study were to assess the remineralizing efficiency of Remin Pro, tricalcium phosphate, and HealOzone by measuring the microhardness of enamel

## Materials and methods

Preparation of samples

A total of 45 extracted premolars for orthodontic reasons from individuals aged 20 to 30 years were taken and immersed in a solution of 0.1% thymol for 15 days. The teeth collected were without anomaly; they were sound teeth, free from any pathology, caries and cracks. Ethical approval was obtained from Maharshi Markandeswar (Deemed to be university), Mullana, Haryana, India, institutional ethics committee with IRB no. 1428/2019.

Infection control protocol for the extracted teeth used in the study

Occupational Safety and Health Administration (OSHA) and Centers for Disease Control and Prevention (CDC) regulations and guidelines were employed in this study to collect, store, sterilize, and handle the removed teeth. Standardized infection protocol was followed for collected extracted teeth for the study. Collected teeth were cleared from calculus, washed then sterilized by immersion in 10% formalin for five days, 5.25% sodium hypochlorite for five days.

Sample size selection

The sample size was estimated using the power calculation α = 0.05 and β = 0.20 with 80% being the power of the study, based on previous findings reported by Karawia et al. [11] and Taneja et al. [13] using the formula n = (zα + zβ )2 o ,2 / d2. Hence required sample size is 36 and we have taken a sample size of 45 for greater accuracy and divided equally into 3 groups with 15 samples each.

Preparation of demineralizing solution

The demineralizing solution was prepared with 2.2 mM calcium chloride (CaCl2.2H2O), 2.2 mM monosodium phosphate (NaH2PO4H2O), and 0.05 M lactic acid. With 50% sodium hydroxide, the pH was raised to 4.5 (Sodium hydroxide [NaOH]). Before and after preparing the solution, the pH was tested with a digital pH meter. Each time the instrument was used to measure pH, it was calibrated using a phosphate buffer solution with a pH of 7.0.

The formula used to group samples

A total of 45 premolars (n = 45) were used for the study and random allocation (randomisation) of teeth was done by lottery method; (n = 15 each): Group A, Group B, and Group C. Then, using an IsoMet diamond disc (Buehler, Illinois, United States), all tooth specimens were sectioned mesiodistally, separating them into two equivalent segments. To smoothen the tooth surface and to improve the accuracy of microhardness measurements, the enamel surface was polished using silicon carbide abrasive sheets ranging from 600 to 3000 grit and finely polished 1-0.25 m water-based diamond cream.

The test groups (n = 20) were further subdivided into two subgroups (n = 10) (demineralized/remineralized) depending on the treatment of the specimen: Group A2 - A2 (a) and A2 (b), Group B2- B2 (a) and B2 (b), and Group C2 - C2 (a) and C2 (b).

Preparation of artificial caries-like lesions

The Amaechi method was used to create white spot lesions. All A2, B2, and C2 test group specimens (n = 60) were submerged in glass containers containing the demineralizing solution and incubated at 37°C for 96 hours to induce artificial caries development, encouraging an active region of demineralization [14]. The solution was changed regularly to avoid the build-up of minerals from demineralization and the resulting pH shift. The samples were rinsed with deionized water for 30 seconds after 96 hours in a demineralizing solution, dried with an air syringe for five seconds, and stored in clean glass containers until further testing.

Remineralizing paste/agents used

Remin Pro® paste was applied with the applicator tip on the tooth surface of samples Group A2 (b) for three minutes for 14 days. ClinproTMTooth Crème (3M ESPE, Maplewood, Minnesota, USA) was applied for three minutes to samples (Group B2 (b) twice a day for 14 days using an applicator tip.

Samples of Group C2 (b) were treated with the ozone-generating HealOzone Unit. The device allowed the application of high-concentration gaseous ozone at 2100 ppm with a flow rate of 615cc/min to the demineralized tooth surface under controlled conditions for the 60s, with the help of an appropriate size sealed cup only once followed by a HealOzone remineralizing paste kit (toothpaste and spray) application, 60s daily for 14 days. During the treatment, ozone needs to be generated on-site because of its molecular instability.

VHN testing

The surface microhardness was measured using VHN testing. The test was performed, in which pyramid-shaped indentations were produced for 20 seconds at a rate of 500-gm load, never close to any edge of the specimen. Three indentations were used to assess the specimen's average microhardness. The VHN of the specimens is determined by the test, and the difference between the baseline, post-demineralization, and remineralization values measured using the identical Vickers indenter settings was used to calculate the change in the VHN for surface microhardness investigations.

Statistical analysis

After the collected data was evaluated with the IBM SPSS Statistics for Windows, Version 21.0 (Released 2012; IBM Corp., Armonk, New York, United States). Data was not normally distributed as tested using the Shapiro-Wilk W-test (p-value >0.05). One-way analysis of variance (ANOVA) (two or more groups) and repeated measures of ANOVA were used for more than two paired readings. Tukey’s post-hoc analysis was done. A level of p<0.05 was measured as statistically considerable.

## Results

**Figure 1:**
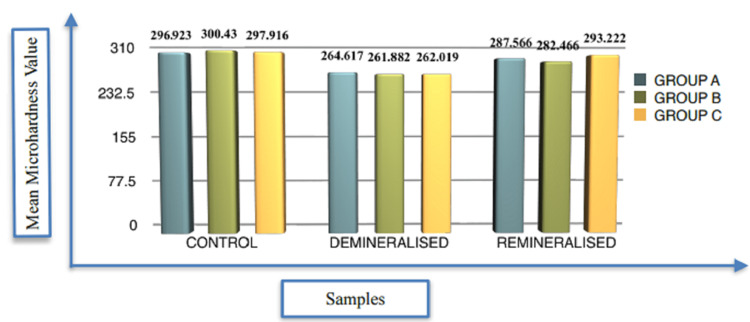
Mean Microhardness Values of Different Groups Used in This Study: Inter Group

It was observed that the control group's mean microhardness values were as follows: group A1 (296.92 kgmm-2), group B1 (300.43 kgmm-2), and group C1 (297.91 kgmm-2). The mean microhardness values for the demineralized groups were as follows: A2a (264.61 kgmm-2), B2b (261.8 kgmm-2), and C2c (262.02 kgmm-2). The mean microhardness values for the remineralized groups were as follows: A2a (287.56 kgmm-2), B2b (282.46 kgmm-2), and C2c (293.22 kgmm-2), with group C2c showing the greatest potential for remineralization compared to groups A2a and B2b. The control group samples had the highest microhardness values, followed by the remineralized and demineralized group samples (Table 1, Figure 1).

**Table 1:** Mean Micro Hardness Values of Different Groups

		Microhardness (kgmm-2)					
		CONTROL		DEMINERALISED		REMINERALISED	
	n	MEAN	SD	MEAN	SD	MEAN	SD
GROUP A	10	296.923	6.108	264.617	8.547	287.566	3.877
GROUP B	10	300.43	4.627	261.882	5.129	282.466	32.782
GROUP C	10	297.916	6.204	262.019	3.127	293.222	5.775

In Table 2, one-way ANOVA was used to assess the remineralization efficacy of various remineralizing materials on the microhardness of enamel. The efficacy was shown to be non-significant in three remineralized groups (Remin Pro, tricalcium phosphate, and HealOzone). The mean microhardness values in the remineralized group were the highest in group C (293.22 kgmm-2), followed by group A (287.5660 kgmm-2), and finally group B (282.4660 kgmm-2).

**Table 2:** Comparison of Efficiency of Different Remineralising Agents on the Micro Hardness of Enamel: Inter Group

ANOVA						
		Sum of Squares	Df	Mean Square	F	Sig.
Remineralised	Between Groups	578.973	2	289.486	0.773	0.471
	Within Groups	10107.634	27	374.357		
	Total	10686.607	29			

From table 3, the control group's mean microhardness was reported to be 298.42 kgmm-2, the demineralized group's 262.83 kgmm-2, and the remineralized group's 284.71 kgmm-2, which are very close to the control group's mean microhardness values. The mean microhardness value of the remineralized group reflects an increase in the microhardness value from the demineralized group value, indicating the remineralizing effectiveness of all the remineralizing agents utilized in this study.

**Table 3:** Descriptive of Comparison of Micro Hardness in Different Groups using One-Way ANOVA Test Descriptive of Comparison of Micro Hardness in Different Groups using One-Way ANOVA Test

	n	Mean	Std. Deviation	Std. Error	95% Confidence Interval for Mean		Min	Max
					Lower Bound	Upper Bound		
CONTROL	30	298.423	5.694	1.03969	296.296	300.5494	290.4	308.8
DEMINERALISED	30	262.839	5.959	1.08799	260.614	265.0645	250.1	274.2
REMINERALISED	30	284.718	18.659	3.40682	277.75	291.6857	270.5	378.5

**Figure 2:**
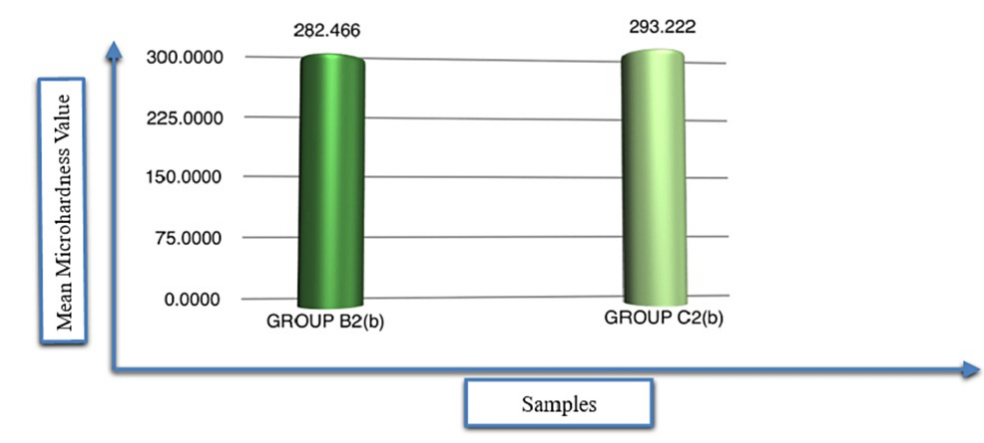
Post-hoc Comparison of Remineralisation Efficiency of Different Remineralising Agents (Group B2 (B) And Group C2 (B)) on the Microhardness of Enamel (Tukey's Test): Inter Group

Post-hoc comparison using Tukey’s test shows statistically non-significant differences between Group B2b and Group C2b. However, Group C2b has a higher microhardness value (293.22 kgmm-2) than Group B2b (282.46 kgmm-2) does (Table 4 and Figure 2).

**Table 4:** Post-hoc Comparison of Remineralisation Efficiency of Different Remineralising Agents (Group B2 (B) And Group C2 (B)) on the Microhardness of Enamel (Tukey's Test): Inter Group

		REMINERALISED (kg mm-2)			
	n	MEAN	SD	Mean difference	P value
GROUP B2 (b)	10	282.666	33.07244	-10.75600	.439
GROUP C2 (b)	10	293.622	1.87535		

## Discussion

As per the guidelines of the Occupational Safety and Health Administration (OSHA) and the Centre for Disease Control and Prevention (CDC) suggestions, this study was carried out after eliminating the risk of any contamination or spread of infection [15]. Balakrishnan et al. stated that the polishing of the specimen is necessary for increased accuracy of microhardness reading as a non-flat surface leads to inaccurate results [16]. A freshly prepared demineralizing solution was used in this study with the final pH adjusted to 4.52 at 37° C with 50% NaOH [17, 18].

The Amaechi protocol was used to induce an artificial caries lesion as there is a consistently increased degree of demineralization at 37°C in comparison to room temperature (20°C) because the calcium and phosphate diffusion coefficients through the enamel and in aqueous solution depend on temperature and increase with increased temperature and an increasing number of days [14].

A remineralization treatment regimen (Remin Pro and Clinpro) was applied two times a day for three minutes as per the manufacturer's recommendations to make it clinically relevant [19,20]. The efficiency was evaluated at 14 days as it takes a minimum of 14 days for any toothpaste/cream to express its desired therapeutic effect [21]. Ozone gas applied for 10 seconds is sufficient to decrease the levels of microorganisms [9,11].

Surface microhardness measurement using the VHN test is an accepted technique for a substrate-like enamel as it has a fine microstructure and a non-homogenous surface prone to cracking. This procedure is comparatively easy, non-destructive, and fast. In this study, three indentations were taken, spaced 20μm apart for microhardness testing to avoid any operational bias [19].

In the present study, the control group showed the higher surface hardness (298.42 kgmm-2) while the demineralized group showed the lowest surface hardness (262.83 kgmm-2) as the acids present in the demineralizing solution impact the intraprismatic and interprismatic parts by degrading the proteins surrounding the enamel rods and crystallites. Thus, some mineral element accompanying the enamel protein is also cleared, decreasing the level of calcium and phosphorus occurring in these areas, which causes microstructural damage with possible changes in microhardness [9]. The results were considered statistically significant and in agreement with studies conducted by Rai et al.., Esfahani et al., and Sandeep et al. [18,22,23].

After 14 days of treatment with remineralizing agents, the mean microhardness of the samples significantly increased compared to the demineralization. Fluoride is one of the main constituents in all remineralizing creams; the hydroxyapatite crystals convert to fluorapatite crystals when they interact with saliva, strengthening the tooth structure and making it resistant to further acid attacks. The presence of calcium fluoride on tooth surfaces acts as a physical barrier, buffering the acids and increasing remineralization [19,24,25].

Remin Pro is more effective in increasing microhardness than is Clinpro, as Remin Pro contains 1450 ppm (61% higher than in the other brands available) of fluoride ions while Clinpro contains only 950 ppm of fluoride [26]. However, there was a difference in opinion in an in-vitro study by Rahul Rao et al .in which Clinpro showed better remineralizing efficiency than Remin Pro. This difference may be due to a change in the mode of application and different incubation time periods [5].

HealOzone showed better remineralizing properties than Remin Pro and Clinpro did. This effect is because the oxidizing property of ozone can eradicate the proteins from the demineralized carious lesions, opening the dentinal tubules, which enhance the remineralization via minerals from saliva or remineralizing agents [27]. Further, ozone helps in the conversion of pyruvic acid to acetate and carbon dioxide, creating an alkaline environment and increasing the diffusion of remineralizing agents, which causes the remineralization of the lesion. Evidence states that only a pH above 5.5 can stimulate remineralization and arrest the progress of the carious lesion [27]. Ozone leads to increased fluoride content in enamel crystals as it oxidizes fluoride into fluorine, which is more active and is easily transported into enamel crystals [28]. Lynch et al. discovered that the zinc present in the kit maintains greater surface-zone porosity, facilitating the diffusion of calcium, phosphate, and fluoride ions into the lesion, allowing more subsurface remineralization to take place. Hence, these remineralized surfaces are more impervious to subsequent decay and acidic challenges [29]. In contrast to HealOzone, tricalcium phosphate has low solubility. Insolubletricalcium phosphate cannot be easily applied, leading to ineffective localization on the tooth surface. It requires acid for solubility to produce ions capable of diffusing into enamel sub-surface lesions [30].

Limitation

Additional research should be undertaken to assess the long-standing outcome of the application of these remineralizing agents. Studies that stimulate in-vivo conditions should be undertaken for conclusive results. Additionally, studies are recommended to measure the depth of lesions under a polarized light microscope before and after treatments.

## Conclusions

Within the limitation of our study, the results of the current investigation concluded that HealOzone is a better remineralizing agent in comparison to f-tricalcium phosphate and Remin Pro. Thus, remineralization procedures can be considered the most preferred and optimal way of regeneration of lost tooth structure, thereby directing the focus of restorative dentistry toward a more conservative approach.

In clinical scenarios, the use of these remineralising agents helps in reducing the risk of dental caries in the population and the initial carious lesions can be reversed clinically.
